# Substance Use among Poisoned Teenage Patients

**Published:** 2019-10

**Authors:** Nasim ZAMANI, Hossein HASSANIAN-MOGHADDAM, Alireza NOROOZI, Mohammad Bagher SABERI ZAFARGHANDI, Ali-Asghar KOLAHI

**Affiliations:** 1. Social Determinants of Health Research Center, Shahid Beheshti University of Medical Sciences, Tehran, Iran; 2. Department of Clinical Toxicology, Loghman-Hakim Hospital, School of Medicine, Shahid Beheshti University of Medical Sciences, Tehran, Iran; 3. School of Advanced Technologies in Medicine, Tehran University of Medical Sciences, Tehran, Iran; 4. Iranian National Center for Addiction Studies, Tehran University of Medical Sciences, Tehran, Iran; 5. Department of Addiction, School of Behavioral Sciences and Mental Health, Tehran Institute of Psychiatry, Iran University of Medical Sciences, Tehran, Iran

**Keywords:** Substance use, Drug abuse, Teenagers, Adolescents, Addiction, Overdose

## Abstract

**Background:**

Substance use is a growing problem in many countries especially among teenagers. We aimed to estimate the prevalence and complications of substance use in teenagers referring to a tertiary hospital following intoxication.

**Methods:**

In a cross-sectional study between 2012 and 2013 in Loghman Hakim Hospital, Tehran, Iran, sixteen substances were checked in teenagers referring due to poisoning. They divided into two groups of young (13 to <16 yr; group 1) and old-teenagers (16 to 19 yr; group 2). History of lifetime substance use and drug use within the week prior to admission were recorded.

**Results:**

Mean (range) age of young teenagers was 14.5±0.6 [13, 15] and 17.7±1.1 [16, 19] in old-teenagers with female predominance. Of 264 teenagers, four in group 1 and 27 in group 2 were admitted due to drug overdose. Six and 69 patients in groups 1 and 2 claimed that they had used some kind of substance in the week prior to admission. Twenty (37%) and 106 (50.5%) patients in the young and old-teenager groups were defined as drug users and rates of unreported substance use were 27.8% (15 cases) and 23.8% (50 cases) respectively. Ninety-six substance users (36.4%) had referred due to poisonings other than recreational intoxication (*P*<.001). Screening toxicological lab data showed significant opioid and sedative exposure in old-teenagers.

**Conclusion:**

It seems young adolescents hide their drug abuse more than old teenagers. Using illicit drugs screening tests may help us to provide hidden rate of abuse in teenagers.

## Introduction

Substance use is a growing and important social problem in many countries including Iran, especially in teenagers and young adults (1, 2). According to WHO, substance use is one of the most challenging behaviors between teenagers, although its rate varies widely among adolescents of different countries with different types of substances (3). The rate of lifetime cannabis use among 15-year-old teenagers, for instance, has been reported to be between 3% in Romania to 34% in Canada (4).

The age of initiation of substance use is generally considered as the age of transition period from mid to late adolescence into early adulthood (5). Teenage years accompany higher risk of substance use causing school dropouts, unsafe sexual activity, accidents, homicides, suicides, and self-injuries (6).

Although no official statistics exist on the prevalence of substance use among teenagers in Iran, some scattered studies have evaluated this social problem in Iranian adolescents. Unfortunately, there are very important drug problems among youth in Iran (7). This rate is increasing in Iranian teenagers as approximately 32%, 30%, and 7% of the high school students in Iran have used alcohol, smoked cigarettes, and used illicit drugs at least once in their lifetime, respectively (3). Almost 6.9% of Tehran high school students have used illicit drugs (8). In a study from Shiraz, 32% of the 10^th^ grade students had consumed alcohol and 2.1% of them had lifetime drug use (9). In Tabriz, 12.7% of the students had used alcohol sometime in their life and 2% had the experience of using drugs (1). However, most of these studies have been performed in the limited setting of one or two grades of high school in limited districts of Tehran or selected provinces. Substance abuse may destroy family cohesion and facilitate suicide ideation in adolescents (10). To our best knowledge, no study has evaluated the teenage substance use rate among vulnerable teenagers with high-risk behaviors.

We aimed to evaluate the prevalence of substance use among teenagers with high–risk behaviors (mainly suicidal attempts) referring to a tertiary poisoning hospital due to intoxication as a part of the substance abuse warning network surveillance system.

## Materials and Methods

This study was a part of the substance abuse warning network surveillance performed between 2012 and 2013 in Loghman Hakim Poison Hospital, Tehran, Iran cross-sectionally (11). All 13-19 yr old teenager patients hospitalized due to poisoning were considered to be included. Those who denied interview or denied to give a urine sample for substance abuse screening test were excluded. After taking history, 16 substances including morphine, methadone, bupernorphine, oxycodone, tramadol, propoxyphene, amphetamine, methamphetamine, 3, 4-ethylenedioxymethamphetamine, cocaine, keta-mine, phencyclidine, tetrahydrocanabinol, benzodiazepines, ethanol, and barbiturates were checked using urine screening immunoassay kits in acutely poisoned patients referring to our emergency department (ED) in two randomly selected days of each week during the study period. Of all patients referred to our center and included, only teenagers (13 to 19 yr) were enrolled into the current study and considered in two different groups of young (13 to <16 yr) and old teenagers (16 to 19 yr) (12).

During this period of time, the trained interviewers visited all ED poisoned patients referred to our center and filled an author-made questionnaire for each single one of them containing data including the patients' demographic characteristics (age, gender, marital status, the people the patients lived with), intent of poisoning (suicidal/accidental/criminal/recreational), name of the drug used, previous history of substance use, history of alcohol or substance use within the week prior to admission, and urine screening tests for opioids, sedatives and hypnotics, stimulants, and hallucinogens. Urine morphine (Rojan®, Cut-off: 300 ng/mL), tramadol (Rojan®, Iran, Cut-off: 100 ng/mL), methadone (W.H.P.M., Cut-off: 300 ng/mL, USA), buprenorphine (W.H.P.M., Cutoff: 12.5 ng/mL, USA), oxycodone (W.H.P.M., Cut-off: 100 ng/ml, USA), propoxyphene (W.H.P.M., Cut-off: 300 ng/mL, USA), amphetamine (W.H.P.M., Cut-off: 1000 ng/mL, USA), methamphetamine (W.H.P.M., Cut-off: 1000 ng/mL, USA), cocaine (W.H.P.M., Cut-off: 300ng/mL, USA), MDMA (W.H.P.M., Cut-off: 1000 ng/mL, USA), hashish (W.H.P.M., Cut-off: 50 ng/mL, USA), ketamine (W.H.P.M., Cut-off: 300 ng/mL, USA), phencyclidine (W.H.P.M., Cut-off: 25 ng/mL, USA), benzodiazepine (W.H.P.M., Cut-off: 300 ng/mL, USA), and barbiturates (W.H.P.M., Cut-off: 300 ng/mL, USA) were checked. Alcohol overdose was checked by quantitative methods on arrival time and/or history of immediate alcohol consumption and clinical manifestations of drunkenness.

Those with ethanol ingestion were, notwithstanding, considered to be alcohol users unless ethanol was consumed to attempt suicide. If the patient had referred due to drug overdose, had not referred due to overdose but mentioned drug or medication use, or had not referred due to overdose and did not give the history of substance use but had positive urine screening tests, he/she was also defined as substance user. The data was entered into SPSS software version 17 (Chicago, IL, USA) and analyzed using descriptive analysis (frequency and percentage), student t-test (mean difference) and further pairwise comparisons for significant variables were checked using Pearson's chi-square test and Fisher's exact test (for categorical data). Confidence interval (CI; 95% accuracy) was presented for significant proportions. A *P*-value less than 0.05 was considered to be statistically significant.

The study meets standards of the ethical conduct of research complied with the World Medical Association Declaration of Helsinki regarding ethical conduct of research involving human subjects and was approved by the Shahid Beheshti University of Medical Sciences Ethics Committee.

## Results

Overall, 264 teenagers were included. Of them, 54 were younger than 16 yr (young teenagers; group 1) and 210 were between 16 and 19 yr (old teenagers; group 2). Patients' demographic characteristics are shown in Table 1.

**Table 1:** Basic characteristics of the studied population

***Variable***		***Group 1 (n=54)***	***Group 2 (n=210)***	**P*-value***
Mean Age (min, max) yr		14.5±.6 (13, 15)	17.7±1.1 (16, 19)	<.001^*^
Female sex n(%)		45 (83.3)	130 (61.9)	0.003 ^†^
Intent n(%)	DSP	44 (81.5)	171 (81.4)	NS
	Accidental	4 (7.4)	9 (4.3)	
	Recreational	4 (7.4)	27 (12.9)	
	Criminal	1 (1.9)	0	
	Unknown	1 (1.9)	1 (0.5)	
Lifetime Suicide history n(%)	DSP	9 (16.7)	48 (22.8)	NS
	Self-mutilation	1 (1.9)	4 (1.9)	
	Self-mutilation + DSP	0	2 (1)	
Job n(%)	Student	45 (83.3)	109 (51.9)	0.01 ^†^
	Solider	0	13 (6.2)	
	Housekeeper	6 (11.1)	50 (23.8)	
	Not employed	1 (1.9)	18 (8.6)	
	Others	2 (3.8)	17 (8.1)	
Marital Status n(%)	Single	50 (94.3)	173 (82.4)	NS
	Married	1 (1.9)	20 (9.5)	
	Fiancé	2 (3.8)	16 (7.6)	
	Divorced	0	1 (0.5)	
Living with n(%)	Parents	43 (81.5)	158 (72.5)	NS
	Mother	5 (9.4)	19 (9)	
	Father	2 (3.8)	4 (1.9)	
	Spouse	1 (1.9)	19 (9)	
	Other relatives	1 (1.9)	5 (2.4)	
	Others	2 (3.8)	5 (2.4)	

* Student t-test,

† Pearson Chi square, DSP=Deliberate self-poisoning

Four cases in group 1 were admitted due to recreational intoxication by ethanol (3 cases) and heroin, whereas in the group 2, 27 cases were recreationally intoxicated by an anabolic steroid i.e. methandienone (1 patient), crack heroin (1 patient), hashish (1 patient), methamphetamine (2 cases), benzodiazepines (4 cases), ethanol (7 cases), and tramadol (16 cases). Five cases had used and been intoxicated by more than one substance (Table 2).

**Table 2:** Number of recreational intoxication, drug use history (based on patients' self-claim and study definition), laboratory results, hidden and total use in groups 1 and 2

***Substance***		***Recreational overdose***	***Substance use history***	***Positive results of lab exam***	***Hidden use (USU)***	***Total***
Opioid n(%)	Group 1	1 (1.9)	2 (3.7)	9 (16.7)	6 (11.1)	8 (14.8)
	Group 2	20 (9.5)	30 (14.3)	67 (31.9)	23 (11)	53 (25.2)
	*P*-value	NS	.035 ^†^	0.027 ^‡^	NS	NS
	95% CI	-	.23 (.05–.99)	2.34 (1.08–5.07)	-	-
Stimulant n(%)	Group 1	0	0	4 (7.4)	4 (7.4)	4 (7.4)
	Group 2	2 (0.8)	2 (0.8)	10 (4.8)	6 (2.9)	8 (3.8)
	*P*-value	NS	NS	NS	NS	NS
Hallucinogen n(%)	Group 1	0	0	2 (3.7)	2 (3.7)	2 (3.7)
	Group 2	1	0	7 (3.3)	2 (1)	2 (1)
	*P*-value	NS	NS	NS	NS	NS
Sedative/hypnotic n(%)	Group 1	0	3 (5.6)	15 (27.8)	7 (13)	9 (16.7)
	Group 2	12 (5.7)	29 (13.8)	89 (42.4)	39 (18.1)	63 (30)
	*P*-value	NS	NS	0.05 ^‡^	NS	NS
	95% CI	-	-	1.91 (.99–3.68)	-	-
Alcohol n(%)	Group 1	3 (5.6)	1 (1.9)	-	-	3 (5.6)
	Group 2	6 (2.8)	7 (3.3)	-	-	12 (5.7)
	*P*-value	NS	NS	-	-	NS
Anabolic steroid n(%)	Group 1	0	0	-	-	0
	Group 2	1 (0.4)	1 (0.4)	-	-	1
	*P*-value	NS	NS	-	-	NS

† Fisher's Exact Test,

‡ Pearson's chi-square test

In a more precise history taken, six patients in the first group and 69 cases in the second group admitted that they had consumed some kind of drug/medication in the week prior to admission (either prescribed by a physician or self-administrated; Table 3). Considering the patients consumed non-prescribed medications, five patients (9.2%) in group 1 and 57 patients in group 2 (27.1%) had used a drug (odds ratio 0.27, 95% CI=0.10-.72, *P*=0.006). Urine screen test results are shown in Table 2. Based on definitions for substance use, 20 (37%) and 106 (50.5%) patients in the first and second groups were substance users, respectively.

**Table 3:** Drugs/medications the teenagers claimed to have been exposed within the week prior to admission

	***Drug category***	***Opioid***	***Stimulant***	***Sedative/hypnotic***	***Hallucinogen***	***Alcohol***	***Anabolic Steroid***
Group 1 (n=54)	ARO (n=4)	2 (50%)	0	0	0	1 (25%)	0
	NRT (n=50)	0	0	3 (6%)	0	0	0
	*P*-value	.004 ^†^	-	NS	-	NS	-
	95% CI	26 (6.68-101.2)	-	-	-	-	-
Group 2 (n=210)	ARO (n=27)	19 (70.4%)	1 (3.7%)	4 (14.8%)	0	3 (11.1%)	1 (3.7%)
	NRT (n=183)	11 (6%)	1 (.5%)	25 (13.7%)	0	4 (2.2%)	0
	*P*-value	<.001 ^‡^	NS	NS	-	.047 ^†^	NS
	95% CI	14.25 (6.87–29.57)	-	-	-	3.62 (1.42–9.23)	-
Total (n=264)	ARO (n=31)	21 (67.7%)	1 (3.2%)	4 (12.9%)	0	4 (12.9%)	1 (3.2)
	NRT (n=233)	11 (4.7%)	1 (.4%)	28 (12%)	0	4 (1.7%)	0
	*P*-value	<.001 ^‡^	NS	NS	-	.008 ^†^	NS
	95% CI	15.22 (7.90–29.34)	-	-	-	4.74 (2.17–10.33)	-

Acute Recreational overdose (ARO), Non-recreational toxicity (NRT),

† Fisher's Exact Test,

‡ Pearson's chi-square test

Based on the number of self-reported substance use and positive test results, hidden (unreported) substance use was estimated to be 27.8% (15 patients) and 23.8% (50 patients) in the first and second groups, respectively (Table 2). Ninety-six substance users (36.4%) had referred due to poisonings other than recreational intoxication (*P*<0.001). Cause of reference in the patients who were substance users but had referred due to poisonings other than recreational intoxication is shown in Table 4. The recreational intoxication and hidden rates of drug use for opioids, stimulants, hallucinogens, and sedative/hypnotics for each gender are shown in Table 5.

**Table 4:** Number of the teenagers who were substance users but had referred due to other causes (n= 96)

***Substance Use***	***Group 1 (n=16)***	***Group 2 (n=80)***	**P-*value***
*Opioids*	6 (37.5%)	34 (42.5%)	NS
*Stimulants*	4 (25%)	7 (8.8%)	NS
*Hallucinogens*	2 (12.5%)	2 (2.5%)	NS
*Sedative/hypnotics*	9 (56.2%)	58 (72.5%)	NS
*Ethanol*	0	5 (6.2%)	NS

**Table 5:** Gender effect on drug use

***Substance***		***Group 1 (n= 54)***			***Group 2 (n= 210)***		

	***Gender***	**Male**	***Female***	**P-*value***	***Male***	***Female***	**P-*value***
Opioids n (%)	recreational overdose	0	1(2.2)	NS	15 (18.8)	1 (.8)	<.001 ^†^
	hidden use (USU)	2 (22.2)	4 (8.9)	NS	5 (6.3)	18 (13.8)	NS
	Total	3 (33.3)	5 (11.1)	NS	25 (31.3)	29 (22.3)	NS
Stimulants n (%)	recreational overdose	0	0	-	1 (1.3)	1 (.8)	NS
	hidden use (USU)	0	4 (8.9)	NS	3 (3.8)	3 (2.3)	NS
	Total	0	4 (8.9)	NS	5 (6.3)	3 (2.3)	NS
Hallucinogens n (%)	recreational overdose	0	0	-	1(1.3)	0	NS
	hidden use (USU)	0	2 (4.4)	NS	0	2 (1.5)	NS
	Total	0	2 (4.4)	NS	0	2 (1.5)	NS
Sedative/hypnotics n (%)	recreational overdose	0	0	-	1 (1.3)	1 (.8)	NS
	hidden use (USU)	1 (4.1)	6 (13.3)	NS	13 (16.3)	26 (20)	NS
	Total users	1 (11.1)	8 (17.8)	NS	24 (30)	39 (30)	NS
Alcohol n (%)	recreational overdose	3 (33.3)	0	.003 ^†^	6 (7.5)	0	.003
	hidden use (USU)	-	-	-	-	-	-
	Total	3 (33.3)	0	.003 ^†^	10 (12.5)	2 (1.5)	.001 ^†^
Total use n (%)	recreational overdose	3 (5.6)	1(1.9)	0.012 ^†^	22 (10.5)	5 (2.4)	<.001 ^‡^
	hidden use (USU)	2 (22.2)	13 (28.9)	NS	15 (18.8)	35 (26.9)	NS
	Total	5 (55.6)	15 (33.3)	NS	52 (65)	54 (41.5)	.001 ^‡^

† Fisher's Exact test,

‡ Pearson's chi-square test

## Discussion

“The center on addiction and substance drug abuse (CASA) in the United States has declared that at least 75% of the high school students in USA have used one or more addictive substances in their life” (13). On the other hand, educational problems, unsafe sexual activities, criminal charges, and self-injuries are probable consequences of substance use in this age group (14). Almost 60% to 80% of the prison and jail inmates and arrestees have been either under the influence of drugs/alcohol during the commission of their crimes or had committed the crime to support a drug addiction (15). This emphasizes the importance of prevention of substance use or drug abuse in prevention of social tragedies.

There are some social, religious, cultural, and economic differences between the Iranian population and other populations in the consumption of addictive substances. This confirms the need for further evaluations in the Iranian teenage population to determine the prevalence of substance use in this country.

The trend of substance use in a poisoning center in Tehran was evaluated and the trend of substance use had significantly increased during the 6-year period of their study (16). However, it did not evaluate this trend in a specific age group. Mehrpour et al. (17) also mentioned such an increasing trend in Iran. However, no study has evaluated substance use in specific age groups such as teenagers except for those from specific provinces.

10.5% of adolescents who committed suicide had a history of substance abuse (18). The history of substance use in the current study was about 28% which is significantly higher than the frequency reported by their studies. This may be due to solely selection of suicide attempters in their study and higher prevalence of substance use during recent years. Considering unreported substance use (USU; including alcohol), our results suggested that risk of substance use was almost 50% in teenagers who referred to us during study period. Although this study was performed on the patients referred to a tertiary poisoning hospital due to intoxication and they were a psychologically special group of patients (they were mainly suicidal), that rate of overt or hidden drug use was still high for that age group. This is especially important with the consideration of the fact that 96 patients (16 in group 1 and 80 in group 2) were actually substance users but had referred due to causes other than overdose showing the great number of adolescents with hidden substance use involvement. This again emphasizes the high risk of vulnerability to drug addiction in our 35-million population of younger than 24 yr (19, 20). The significant difference between young and old teenagers in terms of their substance use is in accordance with the results of the previous studies. However, mid to late adolescence was the time of initiation of drug abuse in many patients (5). This age was early to mid-adolescence in our cases although most of 13- to 16-year-old drug users in our series were actually hidden drug users.

The high rate of USU, especially in the second group and particularly in the female patients for sedative-hypnotics is definitely the main finding of the current study. Sixty-five out of 264 patients (about 25%) were confirmed to be substance users although they denied using any drug or medication. This again confirms the substance use iceberg in the teenagers and mandates urgent interactions by the policy makers to prevent further spread of use among this age group.

Ethanol use was shown to be significantly higher in the male patients of both groups (both *P-*values were 0.003). This shows the fact that risk of overt and hidden alcohol use is significantly higher in the males as mentioned by other previous studies, as well (21, 22). The other point, withdrawn from Table 5, is that the risk of hidden drug use is very near to total number of patients with substance use in group 1 showing the fact that young teenagers are mainly hidden users. Cigarette smoking has been defined as a predisposing factor for using other drugs in the previous studies as regular smokers significantly use drugs more than non-smokers or experimenters (8). We did not evaluate the frequency and severity of dependency on smoking and alcohol in our patients which is definitely a limitation of the current study. However, the main limitation of the current study was that- similar to previous studies- it only evaluated a limited number of teenagers i.e. those referring to our poisoning center due to intoxication. Many of them had attempted suicide which made them a special vulnerable group with higher risk of drug use, dependency, and high-risk behaviors (23). Anyway, they are not representative of whole adolescents. This study shows higher incidence of drug abuse in intoxicated patients, but probably still lower prevalence of substance abuse.

The other potential limitation of the current study was using screening tests as the tool for diagnosis of recent drug use which is definitely less sensitive in comparison to other more advanced techniques of drug determination.

## Conclusion

Substance use is potentially a major health problem in Iranian teenagers at least in specific vulnerable groups. It is forming an iceberg in our country mandating policy makers to determine specific policies to prevent its further spread. Using illicit drugs screening tests may help us to provide hidden rate of abuse in teenagers. Young adolescents hide their drug abuse more than old teenagers. Opioids are the main abused drug among young and old teenagers.

## Ethical considerations

Ethical issues (Including plagiarism, informed consent, misconduct, data fabrication and/or falsification, double publication and/or submission, redundancy, etc.) have been completely observed by the authors.
